# Pilot study: PORTION-O-MAT—a mixed reality solution for investigating perceptual and behavioural abnormalities during food portioning in adolescents with anorexia nervosa

**DOI:** 10.1007/s40519-025-01797-2

**Published:** 2025-11-06

**Authors:** Jessica Gutheil, Oliver Kratz, Martin Diruf, Stefanie Horndasch

**Affiliations:** 1 Department of Child and Adolescent Mental Health, University Hospital Erlangen, Schwabachanlage 6, 91054 Erlangen, Germany; 2 Medical School and University Medical Center OWL, Protestant Hospital of the Bethel Foundation, Department of Child and Adolescent Psychiatry and Psychotherapy, Bielefeld University, Remterweg 13A, 33617 Bielefeld, Germany

**Keywords:** Anorexia nervosa, Mixed reality, Adolescent, Portion size estimation, Food stimuli, Perception bias

## Abstract

**Purpose:**

Anorexia nervosa (AN) is a severe eating disorder characterized by perceptual distortions and restrictive eating behaviours. This pilot study examines portion size estimation in adolescent AN patients using a mixed-reality (MR) approach. The objective is to evaluate the potential of this method for the assessment and treatment of AN, with a particular focus on its ecological validity and its applicability for investigating portion size estimation.

**Methods:**

A total of 30 female participants were recruited: 15 adolescent AN patients and 15 healthy adults as pretest. Participants engaged in a simulated meal assembly task within an MR environment, adjusting portion sizes of virtual food components to match a “typical” meal size (100%). Decision-making patterns and self-reported eating disorder symptoms were recorded. Statistical analyses included descriptive statistics, group comparisons and correlation analysis to examine associations between clinical variables and portion sizes, decision-making time and other decision parameters.

**Results:**

AN patients consistently selected significantly smaller portion sizes than healthy adults, particularly for high-calorie foods. No significant differences were observed in decision-making time or uncertainty indicators.

**Conclusion:**

The findings support the hypothesis that AN patients exhibit altered food perception in the sense that they tend to overestimate the size of visually presented food portions. The MR approach proved effective in simulating meal selection, Future studies should include larger and more diverse samples and incorporate real food intake to further validate these results.

**Supplementary Information:**

The online version contains supplementary material available at 10.1007/s40519-025-01797-2.

## Introduction

AN is a severe mental disorder with significant risks for those affected [1–3]. An increasing number of AN diagnoses has been observed among young people in recent years [3, 4]. The treatment of AN which involves refeeding and consequently the training of adequate meal intake is a critical focus in clinical psychology and medicine. Altered perception of portion sizes is considered a key aspect of the disorder [5–8]. Adolescents with AN face the daily challenge of confronting food or food-related stimuli, which may be perceived as aversive and in conflict with their disorder-related intentions. Appropriate food portioning can thus become a complex and burdensome process.

In the context of eating disorders (ED), changes in attention, information processing, memory, learning, and executive functions are particularly common. Patients exhibit attention biases, indicating altered attentional allocation [9]. This can involve an intensified focus on a specific stimulus (orientation), a reduced ability to shift attention away (distraction), and avoidance of certain stimuli (attention avoidance) [9, 10]. Due to limited cognitive flexibility, a strong cognitive bias often makes it difficult for ED patients to respond selectively and flexibly to anxiety-inducing stimuli [11]. While conclusive data are lacking, it has been suggested that food-related cues may trigger similar attentional distortions in AN patients similar to those elicited by typically anxiety-provoking stimuli [12, 13]. Exposure to food stimuli is often associated with negative emotions in individuals with AN, as they generally rate food images as less pleasant compared to healthy individuals [14–17]. Studies on anxiety have demonstrated a positive correlation between food portion size and both physiological responses (e.g., heart rate, skin conductance) and self-reported anxiety levels [18]. Adolescents with AN exhibit increased pre-meal anxiety and heightened autonomic nervous system activation when exposed to images of larger portion sizes, suggesting that food-related anxiety may play a crucial role in the development and maintenance of AN. Furthermore, research supports the use of computer-based methods to objectively and systematically assess responses to food stimuli [18]. Additional studies have confirmed a positive relationship between portion size and anxiety [5, 19], with findings indicating that the impact of food energy density on anxiety is moderated by portion size [19]. Additionally, ED patients display a heightened focus on details of specific body parts and food attributes, resulting in reduced central coherence. This means they struggle to view acquired information within an overall context [20, 21]. Notably, AN patients often report a high caloric intake even when they objectively consume very little [9]. Adolescents and adults with AN overestimate food size, process food analytically, and resist the height–width illusion—perceptual biases that may impact treatment approaches [22]. They also tend to overestimate portion sizes compared to healthy individuals, though seemingly only for small meals [e.g., 6, 23]. These overestimations are even greater when patients imagine consuming the meals later [9]. However, body size estimations in non-self-referential contexts are comparable to those in control groups, suggesting emotional rather than visual perceptual influences on attention [24]. AN patients also show no systematic sensory-perceptual deficits [25]. These findings largely rule out a general perceptual disorder [9], but provide consistent evidence of nonspecific neuropsychological impairments possibly linked to food-related fear [25–28]. Recent studies have expanded on these findings by investigating the perceptual accuracy of food portions in individuals with AN. Research suggests that AN patients consistently overestimate portion sizes and caloric content compared to healthy controls e.g. [5, 19]. However, the magnitude of these misperceptions varies depending on the context of presentation. When portion sizes are visually presented without immediate consumption, overestimations tend to be more pronounced [6]. In contrast, real-time portioning tasks yield slightly more accurate assessments, though distortions remain present. These results underscore the importance of considering both perceptual and cognitive factors when evaluating food-related behaviours in AN patients [29]. The reward system plays a key role in ED and consists of two components: liking (hedonic pleasure) and wanting (motivation to obtain a reward) [30]. Studies suggest that these aspects are altered in AN. AN patients report reduced liking and wanting, especially for high-calorie foods [13, 31]. They also rate neutral images more negatively after food exposure and show shorter reaction times when choosing between high-calorie foods [32]. The approach-avoidance paradigm suggests that motivationally appealing stimuli are easier to approach than to avoid [9]. However, AN patients demonstrate a reduced tendency to approach food stimuli compared to healthy controls [33, 34]. Findings remain mixed: while evidence suggests a diminished reward response to food, especially high-calorie foods, in AN patients [9], other studies did not find differences between high- and low-calorie foods [33].

A central aspect of this investigation is the confrontation of adolescent AN patients with food stimuli. Several studies have utilized photographs of food for this purpose. Foster and colleagues (2017) attempted to develop food images for use with children and adolescents aged 18 months to 16 years, aiming to use these as an alternative to weighed food diaries—a method in nutrition research where participants document their entire weighed food intake over a defined period of time. This led to the creation of the Young Person’s Food Atlas (YPFA), which showed strong agreement with weighed food diaries, with mostly accurate portion estimates. [35]. Due to a lack of studies accurately assessing food portion perception in AN, a need for more research in this area has been claimed [5]. Traditional methods for assessing portion size perception, such as questionnaires and photographic images, have limitations due to their lack of interactivity. These static approaches fail to fully capture the complexities of real-world food evaluation, where individuals engage with food in a dynamic and multisensory manner. To address this gap, our study employs an MR technique that allows participants to interact with virtual food stimuli in a more immersive and ecologically valid environment. MR technologies are characterized by the combination of virtual and real elements [36]. This approach enhances the accuracy of portion size assessment by integrating perceptual and cognitive factors in real-time, offering new insights into food-related behaviours.

In the context of studies on the treatment of anxiety disorders and phobias, promising results have already been achieved by creating immersive and controlled environments e.g., [37, 38]. The objective of this pilot study was to gain information on the feasibility and characteristics of portion size selection including decision-making patterns of adolescents with AN. To evaluate the potential of the MR method for the assessment and treatment of AN, we asked healthy young adults and adolescents with AN to evaluate our MR device for realism and to estimate portion size. We hypothesized that adolescent patients with AN would estimate significantly smaller portion sizes compared to objective 100% meal standards. Objective 100% meal standards refer to predefined reference meals of standard portion sizes that represent the recommended serving size according to established nutritional guidelines. Furthermore, in an exploratory analysis, given the small sample size, we examined correlations between underestimation and ED symptom severity as well as treatment duration at the time of assessment. We also hypothesized that the decision-making process of adolescent AN patients would differ significantly from that of healthy adults in the pretest. Specifically, we assumed that the overall process would take significantly longer in the AN group and that these patients would show significantly more changes in their selections, whereas pretest participants would more often try out larger portions than their final choice.

We anticipate that, given sufficient ecological validity, this technique may serve not only as a tool for detailed research on systematic biases in portion perception compared to healthy controls and on cognitive distortions affecting portion selection (including associated stress responses), but also as an intervention to normalize portion size estimation through practice-based training in patients with AN.

## Materials and methods

### Participants

This study included 30 female participants, recruited over a period of two months, and divided into two groups: a pretest of 15 healthy adult women and an experimental group of 15 adolescent patients diagnosed with AN. Overall, 16 patients with AN from our clinic were informed about the study. Of these, 15 agreed to participate and obtained consent from their legal guardians. Upon providing consent, all participants completed the full procedure. However, some components were not completed by individual participants, resulting in their partial exclusion from specific analyses.

Participants in the experimental group met the diagnostic criteria for AN or atypical AN according to ICD-10. Recruitment took place at our clinic, with diagnoses confirmed by experienced psychiatrists and psychologists specializing in child and adolescent psychiatry. The German version of CASCAP (Clinical Assessment Schedule for Children and Adolescents with Psychopathology) [39] interview was used for diagnostic assessment. It is a structured interview used to assess a wide range of psychological disorders in children and adolescents.

Exclusion criteria included comorbid active psychosis, severe obsessive–compulsive symptoms that could significantly influence decision-making, acute infectious diseases, pervasive developmental disorders, intellectual disabilities, and pregnancy.

The pretest was conducted with healthy adults recruited from the clinical staff. Apart from screening for the absence of an ED, no additional data were collected, as it primarily served to evaluate technical feasibility and to ensure that portion size estimates were not distorted by the device itself. Accordingly, the pretest was carried out prior to the formal assessment of the experimental group.

The study was conducted on-site at our clinic during inpatient or day clinic treatment. The treatment program was a multidisciplinary program covered by the German health insurance at a child and adolescent psychiatric hospital. An integrative and system-oriented approach aims at recovery of weight, eating behaviour and associated psychological issues such as body image concerns, emotion regulation, social skills, school and family issues. In a behavioral therapy-oriented stepped care approach a weight gain of 0.5–1.0 kg/week and a target weight corresponding to the 25th BMI age percentile are aimed for in accordance with clinical guidelines. Written informed consent was obtained from all participants and, in the case of minors, from their legal guardians. The study received approval from the Ethics Committee of Friedrich-Alexander-Universität Erlangen-Nürnberg.

### Hardware

**Fig. 1:**
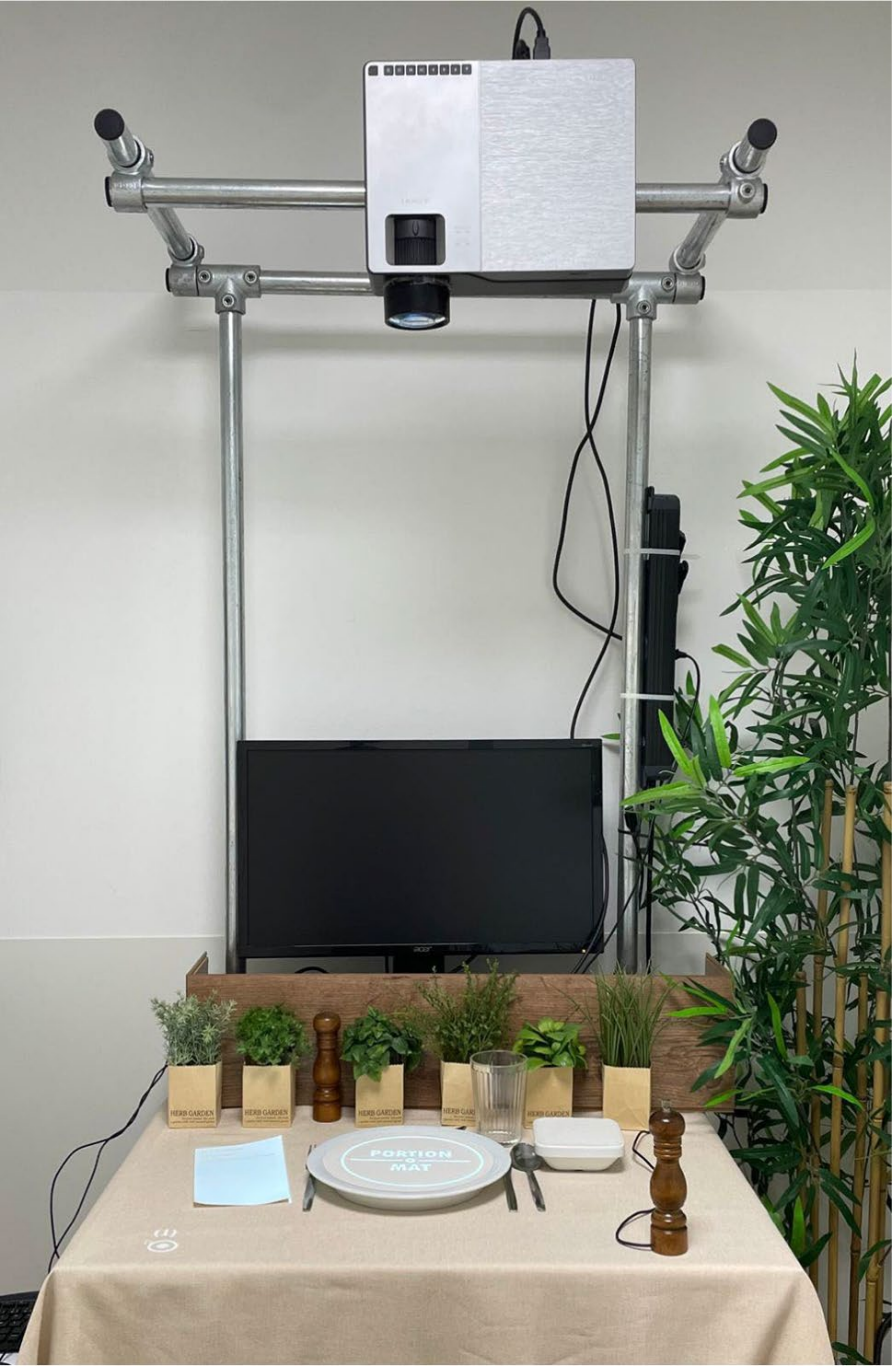
Experimental setup overview

**Fig. 2:**
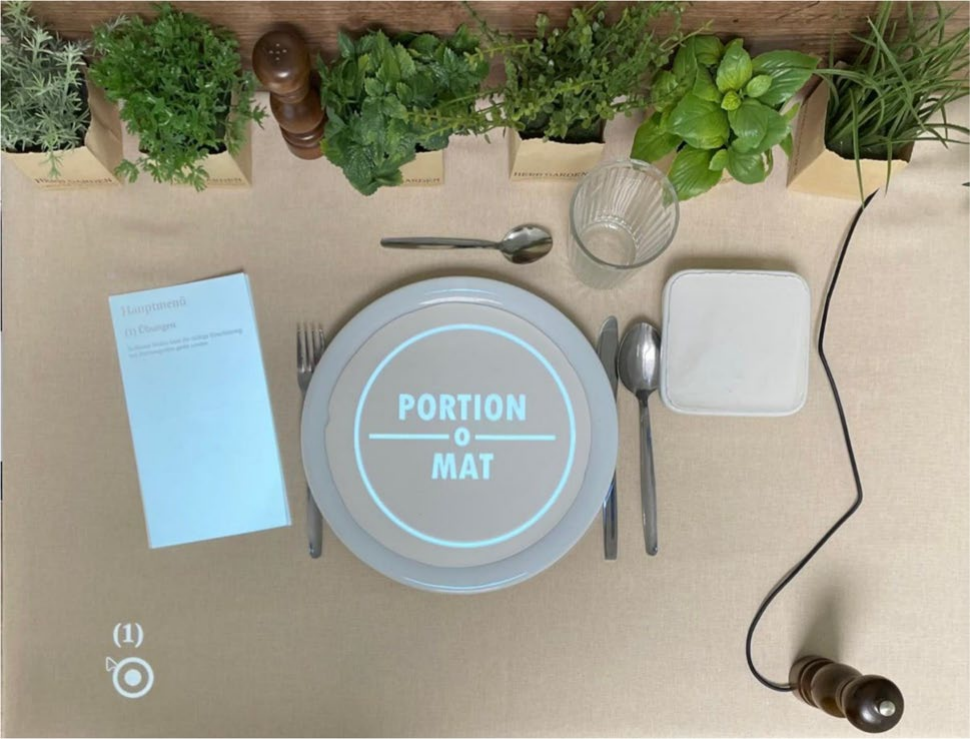
Top view of the “Portion-O-Mat” workspace

The study employed a custom-built MR setup (“Portion-O-Mat”) designed to create an immersive dining simulation. The system consisted of a reconstructed dining table with real tableware, integrated push buttons concealed beneath a tablecloth, and a centrally mounted projector connected to a computer. The computer was operated using a mouse and keyboard, with a display toggle function allowing seamless switching between the monitor and projector. An overview of the hardware can be seen in Fig. 1 and Fig. 2.

The projector displayed meal components onto a plate or salad bowl, alongside meal descriptions and instructions presented on a menu-like card. Participants selected meal components using the push buttons, while portion sizes and quantities were adjusted using a rotary sensor embedded in a pepper mill. To enhance immersion, the push buttons’ functions were dynamically visualized via projected icons. All user interactions, including button presses and rotation inputs, were logged with corresponding timestamps in a database.

### Software

Software development and testing were conducted using XAMPP (Version 8.2.12; Apache Friends), a cross-platform software package providing a local web development environment. XAMPP includes the Apache HTTP (hypertext transfer protocol) Server, the MariaDB database system, and the PHP and Perl programming languages, enabling seamless integration of all required components. The Apache server was accessed via Google Chrome at “https://www.localhost”.

The Web Bluetooth API (application programming interface) in Google Chrome enabled real-time data acquisition from the Polar OH1 pulse sensor. The display application was implemented in PHP, with meal projection based on a preloaded image series. Each image was isolated against a transparent background, allowing individual food components to be layered for realistic composite meal presentations (e.g., spaghetti as a base layer, sauce above it, and Parmesan cheese on top). The portion size of each component could be adjusted independently without affecting the others.

### Stimuli

Five different meals, each composed of three main components and a side salad, were used as experimental stimuli. To control for potential dietary biases, only vegetarian ingredients were included, as individuals with AN often exhibit a preference for vegetarian diets [40]. The selected meals and their components are presented in Table 1.

**Table 1:** Meals with their components

	Component 1	Component 2	Component 3	Component 4
Meal 0: Schnitzel* with French fries	Ketchup	French fries	Schnitzel*	Salad0
Meal 1: Spaghetti Arrabbiata	Spaghetti	Red sauce	Parmesan	Salad1
Meal 2: Gnocchi con Funghi	Mushrooms	Vegetables	Gnocchi	Salad2
Meal 3: Fish sticks* with Potatoes	Remoulade	Potatoes	Fish sticks*	Salad3
Meal 4:Kaiserschmarrn with Apple Sauce	Kaiserschmarrn	Apple sauce	Powdered sugar	Salad4

^*^ To accommodate alternative diets, vegan options were used and explicitly labelled as such

Food components were photographed in incremental portion sizes to prevent participants from inferring a “correct” portion based on image count alone. The increments were standardized within each component, using either weight-based or count-based measurements. The photography setup included a matte blue-painted plate to facilitate image editing, with a camera mounted on a tripod and triggered remotely to ensure image consistency.

**Fig. 3:**
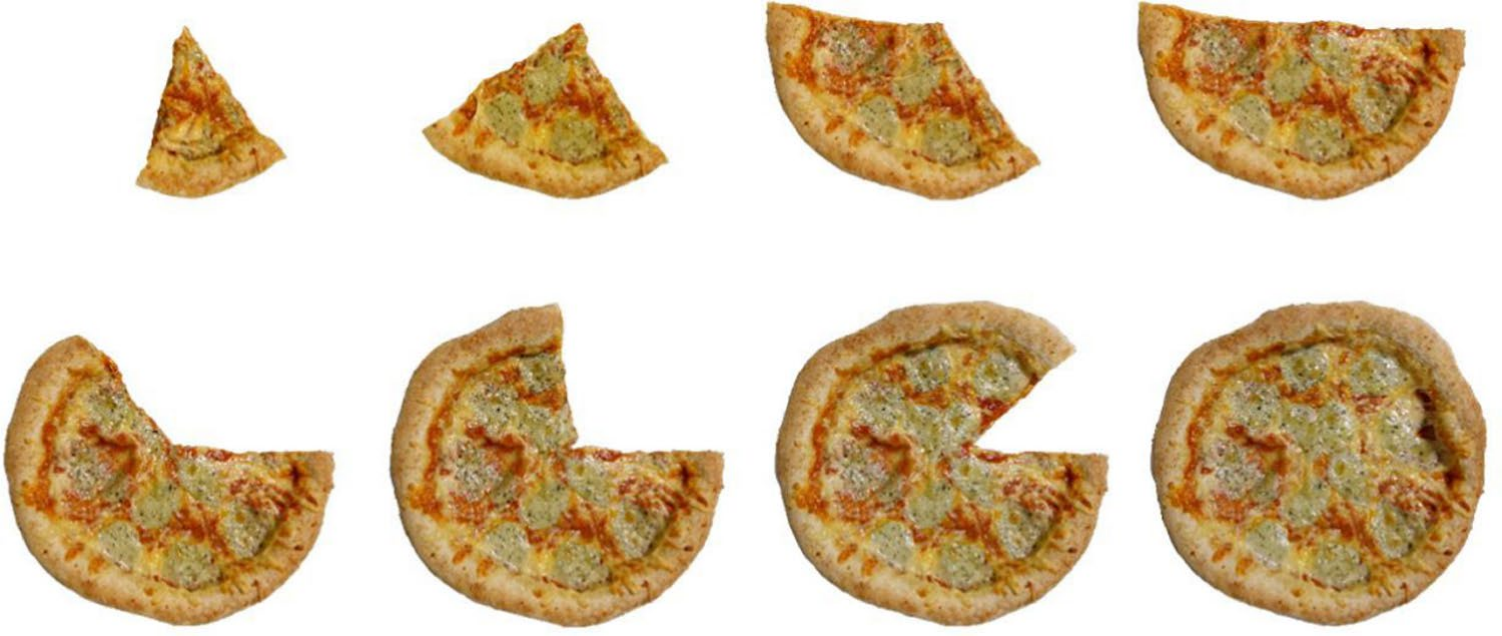
Food stimuli—practice trial

Image processing was performed using GIMP (Gnu Image Manipulation Program, Version 2.10.38). The images were edited into layered files, masked to remove background elements, and converted to a transparent format. All images were resized to 1500 × 1500 pixels. An example of a projected meal component (pizza) is shown in Fig. 3. Image metadata was stored in a MariaDB database for later retrieval in the web application. To enhance the visual realism of the projected components, a CSS-based shadow effect was implemented.

These are the food stimuli we created for the trial run. Portion sizes: 13%, 25%, 38%, 50%, 63%, 75%, 88%, 100%.

Reference portion sizes for each meal component were determined using established nutritional guidelines, including those provided by the German Nutrition Society (Deutsche Gesellschaft für Ernährung, DGE). In addition, the definition of portion sizes in our study was based on the recommendations provided by the Nutrition Therapy Department of the University Hospital Erlangen, as well as on portion size information indicated on product packaging. The DGE recommends an average daily energy intake of approximately 2000 kcal for a balanced diet, distributed across five meals (three main meals and two snacks). The guidelines include specific portion sizes for different food groups. Adolescents aged 15 and older should consume approximately 0.8 g of protein per kilogram of body weight per day, distributed across these five meals. In addition, 50% of the daily energy intake should come from carbohydrates, and 30% should be derived from fats. Meal components were arranged on a plate matching the dimensions of the experimental setup, and photographs were taken at predefined portion size increments. Each image was assigned a percentage relative to the established 100% standard portion. The assignment of variable increments and portion endpoints minimized the risk of participants inferring standard portion sizes based on stepwise progression.

### Procedure

The experimental sessions were scheduled at approximately 10:30 AM, between breakfast and lunch, to minimize the influence of hunger and satiety states on decision-making. Participants were first briefed on the study procedures, after which they completed digital forms and visual analogue scales (VAS) on a tablet as described below. The collected data included demographic information, treatment history, self-reported stress levels, mood, and hunger state.

The following instruction was provided both verbally and in written form: “Welcome to the PORTION-O-MAT. You will encounter five meal tasks in random order. Your goal is to assemble a complete meal using the provided components. The portion size should correspond to a typical adult restaurant serving (100%). You may practice with a trial meal before beginning the actual test.”

Participants completed five meal assembly tasks, selecting portion sizes they deemed appropriate for a full meal (100% portion). The order of meals was randomized. A practice trial was provided before the main experiment. To minimize social stressors, participants completed the main task alone. The program recorded the total session duration, the time to first component selection, and the rotation speed of the pepper mill during portion adjustments.

Upon completing the active session, participants filled out additional self-report measures assessing ED symptoms, perceived realism of the simulation, and food cravings using the short version of the Food Craving Questionnaire (FCQ-T-r) [41]**.** The FCQ-T-r assesses trait food cravings across multiple dimensions and has demonstrated excellent internal consistency (Cronbach’s *α* ≥ 0.93) [41] and good test–retest reliability over a 4-week period. The Eating Disorder Examination Questionnaire (EDE-Q) [42] was used to assess ED psychopathology, including restraint, eating concern, weight concern, and shape concern. The EDE-Q has demonstrated high internal consistency (Cronbach’s *α* = 0.85–0.97) [43] and good test–retest reliability in clinical and non-clinical populations. Additionally, the Eating Attitudes Test (EAT-26) [44] was administered to screen for disordered eating behaviours, with subscales measuring dieting, bulimia, and oral control. The German version (EAT-26D) [45] has shown satisfactory internal consistency (Cronbach’s *α* = 0.83) [46] and is widely used as a screening tool for ED risk. These assessments were placed at the end of the session to prevent biasing performance during the experiment. Participants did not receive immediate feedback on their portion selections to avoid learning effects in potential follow-up trials. However, feedback was provided at the end of the study, supporting the potential application of the MR method for future training interventions.

The assessment of subjectively perceived stress was conducted before the exercise, immediately afterward, and at the end of the study using a 10-point visual analogue scale (“At this moment, I feel …”; 0 = “not stressed at all”; 9 = “extremely stressed”). Patients’ mood was also measured using a 10-point visual analogue scale (“My current mood is …”; 0 = “extremely bad”; 9 = “extremely good”). Similarly, hunger levels were assessed via a 10-point visual analogue scale (“How hungry are you right now?”; 0 = “not hungry at all”; 9 = “very hungry”). To evaluate the subjective realism of the presented food stimuli, another 10-point visual analogue scale was used (“The meals seemed to me …”; 0 = “extremely unrealistic”; 9 = “very realistic”). Particularly regarding the mood VAS, previous findings have shown that its use is meaningful, as the scale—despite its simplicity—yields reliable values, especially in assessing changes over time [47].

### Data analysis

Questionnaire data were collected via SosciSurvey^®^ (SosciSurvey GmbH, Munich) and linked to experimental data using participant ID codes. During testing, all interactions were continuously logged in a database. Data were subsequently exported to Excel for pre-processing and renamed for consistency. Statistical analyses were performed using IBM SPSS Statistics (Version 29.0.1.0; IBM Corporation, New York, USA).

Total questionnaire scores (EDE-Q, EAT-26D, FCQ-T-r) were calculated, and selected variables were recoded where necessary. BMI was computed based on height and weight extracted from patient files. BMI age percentiles were computed according to KiGGS [48]. Interaction times with meal components were aggregated into total session duration, and portion size percentages were averaged across meals for comparative analyses.

To investigate decision-making patterns and potential biases, various measures were analyzed and compared with the results of the pretest data:

- Total meal configuration time: the overall duration required to finalize a complete meal.
- Interaction duration per component: the time spent adjusting individual meal components.
- Decision uncertainty: assessed through the number of directional changes and switches between components during portion adjustments.
- Larger portion trials: examined whether participants tested larger portions before selecting their final meal size.

The hypotheses for this study were formulated prior to data collection. Additionally, the analytic plan was pre-specified, and any analyses that were conducted in an exploratory manner are clearly identified and discussed. Descriptive statistics were computed for categorical variables (n, %) and continuous variables (M, SD). The Shapiro–Wilk test was used to assess normality. Group comparisons and comparisons to reference values were conducted using t-tests. The one-tailed hypothesis test was applied to assess the predicted selection of smaller portion sizes by AN patients. Pearson correlations were computed for normally distributed variables, while Spearman correlations were used for non-normally distributed data. The significance threshold was set at *α* = 0.05. Given the small and unequal sample sizes, effect sizes were calculated using Hedge’s g [49].

## Results

### Participant characteristics

Fifteen female adolescents with AN were included in this pilot study. All participants completed the investigation, and no exclusions were necessary. At the time of testing, 73.3% were undergoing inpatient treatment, while 26.7% were in a day clinic. A summary of the sample characteristics is provided in Table 2.

**Table 2:** Participant characteristics

Variable	Mean	SD
Age (years)	14.80	1.57
BMI (kg/m^2^)	16.93	1.93
BMI age percentile	10.42	10.42
Treatment duration (days)	48.14	30.52
Weight gain (kg)	1.95	1.77
EAT-26D Total Score	31.43	18.41
EDE-Q Total Score	3.30	1.86
EDE-Q Restraint	2.97	2.18
EDE-Q Eating Concern	2.59	1.76
EDE-Q Weight Concern	3.59	1.97
EDE-Q Shape Concern	4.06	2.05
FCQ-T-r Total Score	29.50	13.63

*BMI* Body Mass Index, *EAT-26* Eating Attitudes Test, *EDE-Q* Eating Disorder Examination Questionnaire, *FCQ-T-r* Food Craving Questionnaire (reduced trait version)

### Preliminary analyses

The evaluation of the difference in the portion size estimates from an objectively defined target of 100% in a pretest with healthy female adults showed that nine out of 20 components significantly differed from the target value. Moreover, significant differences were observed in mean portion sizes across entire meals as seen in Table 3. Meals 2 and 3 did not differ significantly from 100%.

**Table 3:** Difference in portion size estimates from an objectively defined target of 100% (pretest)

Meal	Mean portion size (%)	SD	Mean difference	Effect size (g)	*t* value	*p* value
Meal 0	126.32	37.24	26.32	0.67	2.65	0.020
Meal 1	83.54	16.13	− 16.46	− 0.96	− 3.82	0.002
Meal 2	89.94	23.19	− 10.06	− 0.41	− 1.56	0.144
Meal 3	102.71	23.55	2.71	0.11	0.43	0.673
Meal 4	79.71	18.58	− 20.29	− 1.02	− 3.94	< 0.001

Deviation from the 100% target was assessed using a one-sample *t*-test with a test value of 100

To assess the realism of the food stimuli used in the experimental condition, participants with AN rated the stimuli on a scale from 1 to 10, yielding a mean realism score of 6.93 (SD = 1.87). Before the portion selection task, participants also rated their mood and hunger levels (*M* = 5.00, SD = 1.31 and *M* = 2.33, SD = 2.41).

### Portion size selection by participants with AN

One participant was excluded from specific analyses due to missing data. 16 out of 20 components significantly deviated from the test value, demonstrating reduced portion sizes. Significant differences from the test value of 100 were observed in the mean portion sizes averaged across entire meals as you can see in Table 4.

**Table 4:** Difference in portion size estimates from an objectively defined target of 100% (experimental group)

Meal	Mean portion size	SD	Mean difference	Effect size (g)	*t* value	*p* value
Meal0	77.98	14.49	− 22.02	− 1.43	− 5.68	< 0.001
Meal1	46.95	14.80	− 53.05	− 3.39	− 13.89	< 0.001
Meal2	59.46	16.03	− 40.54	− 2.38	− 9.46	< 0.001
Meal3	79.75	19.37	− 20.25	− 0.98	− 3.91	< 0.001
Meal4	52.84	14.45	− 47.16	− 3.07	− 12.22	< 0.001

Deviation from the 100% target was assessed using a one-sample *t*-test with a test value of 100

In addition to this analysis, a group comparison was conducted comparing portion selection between the pretest and the experimental group. It revealed significant differences for all meals as seen in Table 5.

**Table 5:** Comparison of portion choices between pretest and experimental group

Meal	*t* value	Effect size (g)	*p* value
Meal0	− 4.53	− 1.66	< 0.001
Meal1	− 6.37	− 2.30	< 0.001
Meal2	− 4.00	− 1.49	< 0.001
Meal3	− 2.82	− 1.03	0.005
Meal4	− 4.21	− 1.57	< 0.001

Group comparisons were performed using *t*-test

### Influence of participant characteristics

Correlation analyses examined the relationship between treatment-related factors and portion size selection. A significant positive correlation was found between treatment duration and portion size selection for Meal0 (*r*(12) = 0.60, *p* = 0.023). However, no significant associations were observed for other meals or for total scores of the EAT-26D, EDE-Q, and FCQ-T-R questionnaires. Furthermore, BMI, mood, hunger levels, perception of realism, and prior treatment duration were not significantly correlated with portion size selection. Subgroup comparisons based on treatment setting (inpatient vs. day clinic) also yielded no significant differences.

### Decision-making characteristics

The duration of participant interactions with the portion selection interface was recorded for each component and aggregated across entire meals. Three participants were excluded from some analyses due to missing data. No significant group differences between AN patients and the pretest sample were found regarding total meal selection time. However, significant differences emerged only for the specific component Ketchup (*t*(16.07) =  − 2.81, *p* = 0.013). An overview can be seen in supplement A.

To assess decision-making behaviour, the frequency of directional changes (increases or decreases in portion size) and the number of component switches was analyzed (Table 6). These variables did not follow a normal distribution, and revealed no significant differences between groups regarding total directional changes (*U* = 65.50, *p* = 0.220) or total component switches (*U* = 86.50, *p* = 0.830).

**Table 6:** Comparison in decision making behaviour between pretest and experimental group

	AN		Pretest		Comparison AN x Pretest*	
	Mean	SD	Mean	SD	U	*p*
Directional changes	10.64	7.29	18.31	15.00	65.50	0.220
Component switches	3.93	4.30	4.54	4.74	86.50	0.830
Larger Portion Sizes	4.93	3.41	6.08	3.73	74.00	0.430

Group comparisons were performed using Mann–Whitney-*U*-Test

An analysis of the number of times participants selected a portion size larger than their final choice revealed no significant difference between the groups (experimental group: *M* = 4.93, SD = 3.41; pretest group: *M* = 6.08, SD = 3.73; *U* = 74.00, *p* = 0.430).

## Discussion

### General discussion

The present study examined portioning decisions among adolescents with AN compared to a pretest in healthy participants using a MR approach. It demonstrated that adolescents with AN consistently selected smaller portion sizes compared to healthy participants, while decision times did not differ significantly between groups. No significant effects of variables such as treatment duration, BMI, mood, hunger, or perceived realism on portion size selection were observed, and ED symptomatology from the questionnaires also did not reveal a clear effect.

The high rate of consent and low discontinuation rate during the study indicate a high degree of acceptance among patients with AN despite the possibly aversive food-related content. Similarly, all female adult participants from the pilot study, recruited simultaneously with the AN group, who were approached agreed to participate. One healthy participant was excluded from the analysis due to repeated interruptions during meal size configuration. All others completed the study without any issues which indicates a high degree of feasibility. The integration of MR technology into this study represents a key methodological advancement. A recent review observed a “highly experimental character and a certain laboratory atmosphere” in most studies on food portioning in AN [5]. Participants in the current experiment rated the visual stimuli as sufficiently realistic, indicating that the MR environment effectively simulated relevant features of real meal scenarios. This realism is crucial for external validity, as it enhances the ecological relevance of the findings. Nonetheless, it remains unclear whether virtual meal presentation elicits the same emotional, physiological, and motivational responses as actual food exposure. Further research is needed to directly compare virtual and real-life food interactions in terms of neural and behavioural outcomes.

The results indicate significant differences in portion selection between groups, with AN patients consistently choosing smaller portions than healthy adults. These findings align with previous research suggesting that individuals with AN systematically underestimate portion sizes and overestimate caloric content [6–9].

A key finding of the study is that AN patients demonstrated a significant reduction in portion size selection compared to the control group. This could be explained by increased anxiety toward specific foods and a distorted cognitive evaluation of food intake [13, 31]. These results align with neurobiological models that attribute altered food-related decision-making in AN to dysfunctions in the reward circuitry [30], which may skew food evaluation processes and reduce the hedonic value of eating.

Interestingly, the study found no significant differences in decision times between the groups. This suggests that the selection of reduced portion sizes by AN patients is not due to increased hesitation or uncertainty but rather to a consistent, albeit distorted, food evaluation. It should nevertheless be considered that the reasons for the duration of meal configuration may differ between groups. The healthy participants showed a noticeable playful interest in the “Portion-o-Mat”, meaning that the total duration does not accurately reflect decision speed, whereas this assumption is more applicable to the AN group. Additionally, the analysis of decision patterns (e.g., changes in portion size during selection) did not reveal significant differences, indicating that the selection process itself may not be primarily driven by impulsive factors but rather by deeply ingrained cognitive biases. It should be considered that measures such as “Total Meal Configuration Time” and “Interaction Duration per Component” may be confounded by mechanisms other than decision-making, such as food avoidance or food-related phobia.

The observed correlation between hospitalization treatment duration and increased portion size in one meal condition suggests a potential normalization effect with therapeutic progress. Although tentative, this points to the value of longitudinal monitoring of food evaluation behaviour. Therapeutic interventions may, over time, help recalibrate distorted food perceptions—particularly when coupled with exposure to realistic meal situations, as enabled by the MR setup. The results confirm that the visual stimuli in the MR setup were perceived as realistic, strengthening the external validity of the method. However, it remains unclear whether virtual representations of meals evoke the same emotional and physiological responses as direct confrontation with real food.

Based on our findings and those of previous studies [5, 7, 8], a potential therapeutic approach could involve regular visual exposure to progressively larger food portions, with ongoing monitoring of changes in portion size estimation throughout treatment, aiming to recalibrate patients’ visual perception of what constitutes a “normal” portion size.

Despite the study’s strengths, several limitations must be acknowledged. First, the relatively small sample size—constrained by the clinical context and the novelty of the technology—limits generalizability. Future studies should recruit larger and more diverse samples to enhance statistical power and ensure a broader representation of the AN population. This would also allow for more robust outlier detection and subgroup analyses.

Moreover, stress-related responses to the task were not systematically assessed. Since food exposure may trigger anxiety in AN patients [6], future studies should incorporate physiological (e.g., heart rate, salivary cortisol) and subjective measures of stress. These data would provide valuable insights into the emotional underpinnings of portion-selection behaviour.

The timing of assessment is also a relevant factor. Some participants were near the end of their treatment and may have already undergone therapeutic interventions that influenced their behaviour. Future research should prioritize earlier stages of treatment and include repeated measures across the treatment timeline to examine intra-individual change. A longitudinal design would be particularly beneficial in assessing whether perceptual biases shift as patients progress through therapy. Recent findings by Pasi et al. emphasize that perceptual misjudgements of portion size in individuals with AN persist across various stages of illness and recovery, suggesting a relatively stable cognitive bias rather than a transient symptom [7]. This highlights the importance of studying a broader spectrum of illness severity and recovery stages in future research, to better capture the persistence and variability of these distortions.

Regarding the control group, the current pretest was conducted with healthy adults. To enhance developmental comparability, future research should include healthy adolescents matched in age and educational background with the clinical group. These control participants should also complete standardized measures of ED symptomatology to rule out subclinical pathology. The analyses focussing on the comparison between the experimental group and the pretest must be regarded as highly exploratory.

Some pretest participants experienced technical difficulties and approached the task with a more playful attitude. This highlights the importance of standardizing instructions and improving technical reliability. As MR technology continues to evolve, improvements in interface responsiveness, visual quality, and environmental realism are expected. Enhancing the immersive quality of the task—for example, through the inclusion of food odours, ambient kitchen sounds, or lighting adjustments—could further simulate real-life eating scenarios and deepen emotional engagement. Even though an influence of intent-to-eat could not be demonstrated by Pasi and colleagues, it remains another important factor that should be re-examined in future studies [7].

Improvements to the food database are also warranted. Expanding the range of meal components—particularly desserts and mixed dishes—as well as the exact determination of caloric content would allow for a more comprehensive analysis of food-related decision-making. As Dörsam and colleagues have already demonstrated, different food characteristics have varying impacts on perceptual distortions [5]. Accordingly future work should further investigate how variables such as energy density, palatability, and individual hunger levels influence portioning behaviour. Furthermore, creating a broader spectrum of meal choices for the Portion-O-Mat could allow researchers to compare decisions involving sweet vs. savoury meals and shed light on food-type-specific avoidance tendencies.

In addition, correlating portioning behaviour with subscale scores from validated ED questionnaires (e.g., drive for thinness, fear of weight gain) could help identify symptom-specific cognitive distortions. More detailed metrics—such as time to first interaction, speed of increasing or decreasing portions, and the number of exploratory actions—may provide a nuanced understanding of the decision process.

Finally, post-task evaluations in which participants are asked whether they could realistically consume the portioned meal—or what percentage of it they believe they could eat—could bridge the gap between simulated decisions and real-world behaviour. A particularly promising avenue would be to examine whether portioning behaviour changes when participants expect to eat the selected meal after the task, thereby introducing motivational relevance and accountability into the decision-making process.

### Strength and limits

This study’s main strength is the integration of MR technology as a methodological innovation, enhancing validity by providing a realistic simulation of meal scenarios. The high consent rate and minimal dropout among adolescents with AN, despite the aversive nature of food-related tasks, underline the feasibility and acceptability of this approach in clinical populations. Furthermore, the consistent group differences in portion size selection, aligned with prior research, support the construct validity of the paradigm.

However, important limitations must be acknowledged. The small, clinical-specific sample limits generalizability, and the use of adult pretest participants rather than age-matched controls reduces developmental comparability. Therefore, analyses regarding the pretest could only be conducted at a highly exploratory level. Additionally, technical issues and varying levels of playful task engagement in the pretest group further highlight the need for more standardized instructions and continued refinement of the MR system.

### What is already known on this subject?

Prior research has shown that patients with AN often overestimate portion sizes and caloric content compared to healthy controls, but findings have been inconsistent. These perceptual biases appear to be linked less to general sensory deficits and more to food-related anxiety, attentional biases, and altered reward processing, as patients consistently report reduced liking and wanting for high-calorie foods and demonstrate diminished approach tendencies toward foods. While traditional assessment methods such as questionnaires and static food images have provided valuable insights, they lack ecological validity, highlighting the need for interactive and immersive approaches like MR, which have shown promise in related fields such as anxiety and phobia treatment.

### What your study adds?

This study shows that an MR approach is both feasible and acceptable for adolescents with AN and can detect systematic biases in portion size estimation. These findings suggest that MR technology may be a valuable tool for future research and could inform the development of novel therapeutic interventions targeting distorted food-related decision-making.

## Conclusion

This pilot evaluation of an MR technology shows a high degree of feasibility and acceptability of this method in young female participants. Its preliminary results contribute novel insights into the food-related decision-making of individuals with AN. The findings underscore the role of cognitive distortions in portion selection and highlight the potential of MR technology to provide ecologically valid, yet controlled, assessment environments. Continued refinement of this approach, paired with larger and longitudinal studies, will be essential for understanding the mechanisms underlying maladaptive eating behaviours and developing targeted, evidence-based interventions.

## Supplementary Information

Below is the link to the electronic supplementary material.

Supplementary Material 1.

## Data Availability

The datasets generated during and/or analysed during the current study are available from the corresponding author on reasonable request.
