# Supplementary material for: Pilot study: PORTION-O-MAT—a mixed reality solution for investigating perceptual and behavioural abnormalities during food portioning in adolescents with anorexia nervosa
Source: Eat Weight Disord. 2025 Nov 6;30(1):84. doi: 10.1007/s40519-025-01797-2 (PMC12592256; doi:10.1007/s40519-025-01797-2)
Supplement: Supplementary file 1 — Supplementary Material 1. [file 40519_2025_1797_MOESM1_ESM.docx]

Supplements

Supplement A

**Comparison of Decision-Making Characteristics between Pretest and Experimental Group**

|  | AN | | Pretest | | Comparison AN x Pretest* | | |
| --- | --- | --- | --- | --- | --- | --- | --- |
|  | Mean | SD | Mean | SD | t | df | p |
| **Total Meal Configuration Time** | 210.16 | 57.42 | 236.60 | 62.10 | -1.191 | 27 | .244 |
| **Interaction Duration Per Meal and Component (sec):** |  |  |  |  |  |  |  |
| Duration Meal0 | 44.14 | 20.28 | 56.19 | 28.28 | -1.327 | 27 | .196 |
| Duration Ketchup | 7.84 | 2.65 | 13.73 | 7.41 | -2.811 | 16.07 | .013 |
| Duration French Fries | 11.08 | 6.57 | 13.70 | 9.16 | -.888 | 27 | .383 |
| Duration Schnitzel | 12.67 | 7.13 | 13.71 | 10.05 | -.324 | 27 | .749 |
| Duration Salad0 | 9.36 | 4.76 | 8.94 | 4.29 | .245 | 26 | .808 |
|  |  |  |  |  |  |  |  |
| Duration Meal1 | 38.62 | 14.37 | 34.01 | 8.93 | 1.030 | 27 | .312 |
| Duration Spaghetti | 12.04 | 7.61 | 10.80 | 4.35 | .530 | 27 | .600 |
| Duration Red Sauce | 7.33 | 3.46 | 7.52 | 3.46 | -.142 | 27 | .888 |
| Duration Parmesan | 6.38 | 3.08 | 5.30 | 2.09 | 1.097 | 27 | .282 |
| Duration Salad1 | 10.13 | 4.00 | 7.85 | 1.94 | 1.973 | 20.545 | .062 |
|  |  |  |  |  |  |  |  |
| Duration Meal2 | 41.47 | 13.48 | 46.91 | 16.07 | -.989 | 27 | .332 |
| Duration Mushrooms | 11.57 | 4.79 | 14.59 | 7.67 | -1.279 | 27 | .212 |
| Duration Vegetables | 8.11 | 4.57 | 8.59 | 4.49 | -.289 | 27 | .775 |
| Duration Gnocchi | 10.92 | 4.94 | 12.84 | 5.53 | -.966 | 26 | .343 |
| Duration Salad2 | 8.24 | 2.58 | 7.56 | 2.60 | .687 | 25 | .499 |
|  |  |  |  |  |  |  |  |
| Duration Meal3 | 43.06 | 12.28 | 53.21 | 22.11 | -1.542 | 27 | .135 |
| Duration Remoulade | 9.65 | 3.53 | 9.99 | 4.66 | -.220 | 27 | .827 |
| Duration Potatoes | 11.88 | 4.77 | 13.17 | 5.95 | -.648 | 27 | .523 |
| Duration Fish Sticks | 10.08 | 3.38 | 14.88 | 12.28 | -1.411 | 14.838 | .179 |
| Duration Salad3 | 8.75 | 3.11 | 10.87 | 5.11 | -1.328 | 26 | .196 |
|  |  |  |  |  |  |  |  |
| Duration Meal4 | 42.87 | 18.18 | 46.29 | 15.89 | -.538 | 27 | .595 |
| Duration Kaiserschmarrn | 15.40 | 12.67 | 18.20 | 7.62 | -.713 | 27 | .482 |
| Duration Apple Sauce | 9.74 | 3.15 | 8.73 | 3.00 | .889 | 27 | .382 |
| Duration Powdered Sugar | 6.32 | 1.67 | 5965 | 2.53 | .453 | 27 | .654 |
| Duration Salad4 | 7.41 | 3.23 | 10.21 | 4.78 | -1.799 | 25 | .084 |

*Group comparisons were done using independent samples t-test*
